# Statistics of the Hospitals and the Medical Profession in Paris

**Published:** 1849-10

**Authors:** 


*Statistics of the Hospitals and the Medical Profession in Paris.*

The administration of the Parisian hospitals employs 2,500 individuals, and possesses a budget of from fifteen to sixteen million francs. There are fifteen hospitals, furnishing 7174 beds, and receiving 90,000 patients per annum. Besides these, there are four large hospices. and seven retreats for 8000 aged and infirm persons. More than 100,000 receive *secours a domicile;* and above 25,000 foundlings are provided for. The following are the names and number of beds of the various hospitals:

| General Hospitals. | | Special Hospitals. | |
|---|---|---|---|
| Hotel-Dieu, | 810 beds. | St. Louis, | 825 beds. |
| St. Marguerite, | 300 do | Du Midi, | 300 do |
| La Pitie, | 621 do | De l'Orsine, | 300 do |
| La Charite, | 494 do | Enfans Trouves, | 600 do |
| St. Antoine, | 320 do | Maison d'Accouchement, | 514 do |
| Necker, | 329 do | Maison des Cliniques, | 120 do |
| Cochin, | 325 do | Maison de Sante St. Denis, | 150 do |
| Beaujon, | 438 do | | |
| Bon Secours, | 324 do | | |
| De la Republique | 600 do | | |

A year or two since, some of the Parisian medical journals, alarmed at the constant increase of the numbers of the profession, called aloud for some legislative means of repression. The number of qualified doctors of medicine steadily increased from 1090 in 1833, to 1442 in 1847; added to which, there were, in this last year, 175 of the lower qualified practitioners, termed *officiers de sante*, giving 1617 legalized practitioners for little more than a million souls (including hospitals, garrison, and other unremunerating bodies); while in London we had, at the same period, but 2500 regular practitioners for our two millions. Moreover, the midwives are, in Paris a numerous body (480 in 1847), absorbing much remunerative practice, which, in London, falls to the practitioner. Then, again, classes of persons there resort to hospitals, who here pay for their attendance. If the remaining patients had been equally divided among the 1617 regular practitioners, it was calculated that 150 per annum would fall to each,—the charge for visits being 5, 3, 2 francs, or even less, and no bad debts being recoverable at the expiration of a year. One of the effects of the revolution has been, to produce at least a considerable temporary diminution of the numbers. Thus, at the commencement of the present year, there were but 1389, instead of 1442 doctors; the whole number of qualified practitioners diminishing from 1617 to 1555, and that of midwives, from 480 in 1847, to 385. The *pharmaciens* are the only portion of the body medical who have held their ground, their numbers being 345 in 1847, and 363 in 1849.—*British and Foreign Med. Review.*

